# Defining Pre-Clinical Psoriatic Arthritis in an Integrated Dermato-Rheumatology Environment

**DOI:** 10.3390/jcm9103262

**Published:** 2020-10-12

**Authors:** Laura Savage, Ilaria Tinazzi, Alen Zabotti, Philip M. Laws, Miriam Wittmann, Dennis McGonagle

**Affiliations:** 1 Leeds Institute of Rheumatic and Musculoskeletal Medicine, University of Leeds, Leeds LS7 4SA, UK; laura.savage4@nhs.net (L.S.); philip.laws2@nhs.net (P.M.L.); m.wittmann@leeds.ac.uk (M.W.); 2 Chapel Allerton Hospital, The Leeds Teaching Hospitals NHS Trust, Leeds LS7 4SA, UK; 3 Rheumatology Unit, IRCCS Sacro Cuore Don Calabria, 37024 Negrar, Italy; ilariatinazzi@yahoo.it; 4 Department of Medical and Biological Sciences, Rheumatology Clinic, University Hospital Santa Maria della Misericordia, 33100 Udine, Italy; zabottialen@gmail.com

**Keywords:** psoriasis, psoriatic arthritis, integrated dermato-rheumatology clinic, psoriatic arthralgia, pre-clinical psoriatic arthritis

## Abstract

In excess of three quarters of patients with psoriatic arthritis (PsA) have preceding psoriasis (PsO), which offers a clinical biomarker for the recognition of early PsA. Numerous surveys have shown a remarkably high frequency of clinically occult musculoskeletal symptoms in psoriasis patients. Imaging studies, particularly ultrasound, show a high prevalence of subclinical enthesitis and other inflammatory changes in psoriasis subjects. Since a serum biomarker, such as the case of anti-citrullinated protein antibodies (ACPA) in rheumatoid arthritis, neither exists nor seems biologically plausible at this point, this article explores how integration of rheumatological and dermatological assessment can be facilitated for the early recognition of potential PsA. Given that scalp disease is a PsA predictor, but may be managed in the community, then a particular need to access this group is needed. An integrated approach between rheumatology and dermatology can involve joint clinics, parallel clinics with discussion of relevant cases or virtual contact between specialties. Early therapy evaluation and integrated strategies have considerable implications for minimizing suffering and joint damage in PsA.

## 1. Introduction

Psoriasis (PsO) constitutes a sizeable proportion of the dermatologist’s workload with an estimated prevalence in the general population of 2%. Psoriatic arthritis (PsA) is an inflammatory, seronegative spondyloarthropathy affecting the peripheral and/or axial joints and develops in between 6% and 42% of patients with PsO [1,2,3,4,5,6,7,8]. Together, manifestations of both are amalgamated under the term “psoriatic disease” (PD). Six out of every seven patients with PD will develop cutaneous disease first [9], with a typical time delay of anywhere between seven and twelve years [10]. PsA affects men and women equally, and usually begins in the fourth decade [1,4]. Approximately half of patients affected by PsA develop erosive joint disease [11], which if left untreated leads to marked functional limitation, pain, and disability [4]. Rapid diagnosis of aggressive PsA and appropriate therapeutic intervention is therefore of paramount importance.

The understanding of the immunopathogenesis of PsA has been transformed in the past two decades. Whilst initially conceptualized largely in relationship to an autoimmune disease [1,4], attacking the skin on one hand and the synovial membrane (leading to joint swelling) on the other, it has emerged that entheseal inflammation may be the key feature in early PsA [12,13]. This understanding has been underpinned by musculoskeletal imaging, including ultrasound and MRI in both PsA and in PsO patients where a high prevalence of subclinical entheseal inflammation has been reported in patients with skin disease alone [14,15,16].

The emergence of an effective armamentarium of biological drugs and other molecules for the treatment of both PsO and PsA means that it now might be possible to prevent arthritis evolution. The widespread use of biologic therapy for PsO may be reducing the incidence of PsA, but further work is needed to evaluate this possibility. We have recently published imaging studies in psoriasis that support this, since subclinical active enthesitis in psoriasis may regress under therapy [17]. These combined factors are leading to a new paradigm that is built around the concept that the recognition of subclinical arthropathy is possible and that PsA could even be preventable, potentially without any additional costs or risks, since patients often require systemic therapy for the severity of skin disease alone [18]. This article highlights the ways in which dermatological and rheumatological practice can interact and evolve to realize these goals.

## 2. From PsO to PsA in the Dermatology-Rheumatology Combined Clinic

### 2.1. The Pre-Clinical Phases of Psoriatic Arthritis

It has recently been proposed that there are three clinical phases after PsO onset and before clinical PsA development [19]. Two initial asymptomatic phases, at first with aberrant activation of the immune system which may originate from the skin, intestinal mucosa, or the entheses and then with subclinical signs of musculoskeletal inflammation or with soluble biomarkers (e.g., ESR, CRP). Lastly, a prodromal symptomatic transition phase may exist characterized by nonspecific musculoskeletal pain, recently defined as psoriatic arthralgia, and fatigue [8,20]. This prodromal phase can be difficult to identify due to non-specific symptoms and possible PsA disease mimics, such as incipient or concomitant osteoarthritis, fibromyalgia, or chronic pain related to psoriatic comorbidities [21,22,23]. There are preliminary evidences that sonographic determined enthesitis might be associated with subsequent clinical disease evolution [20,24,25], but these results are preliminary and require further validation. However, as discussed below, there is a tremendous need to study PsO phenotypes themselves more closely and to use imaging in the context of psoriasis patterns to better define the pre-clincal phases of PsA. In this regard, PsO phenotypes characterized by nail and scalp involvement seem to have higher risk for the subsequent development of PsA [26] and were used for preliminary studies on PsA interception [27]. Given the large volume of patients with PsO in the dermatology clinic, close collaboration between Dermatology and Rheumatology is essential for academic studies and to optimize clinical care.

### 2.2. The Need for Early Evaluation and Intervention

The impetus for the recognition of early PsA and its effective treatment stems from rheumatoid arthritis (RA) [28,29], where early disease recognition and prompt therapy initiation minimizes joint damage, maintains patients in work, and reduces long-term complications. However, unlike RA, a reliable serum biomarker, such as anti-citrullinated protein antibodies (ACPA), does not exist for PsA [30,31]. Given that the majority of PsA patients either have preceding or contemporaneous PsO [1,4], this clinical cutaneous biomarker therefore places dermatologists at an ideal juncture for the early recognition of PsA (Table 1). This is all the more relevant now that therapies that treat both skin and joint disease exist, and that dermatologists increasingly recognize the systemic nature of PsO, including its musculoskeletal and cardiovascular manifestations.

While dermatologists are competent in the management of psoriatic skin disease, many are not sufficiently familiar with the diagnosis, treatment, and referral criteria of co-existent PsA. Despite the advent of sensitive imaging techniques that can detect pre-symptomatic arthritis, within the context of a busy dermatology clinic, imaging of every patient with PsO may not be clinically or economically appropriate. Coupled with the lack of a reliable biomarker, this has led to the development of several PsA screening tools with variable sensitivity and specificity, including the Psoriasis Epidemiology Screening Tool (PEST) [32], Toronto Psoriatic Arthritis Screen (ToPAS) [33], the Psoriatic Arthritis Screening and Evaluation (PASE) [34], and Early Arthritis in Psoriasis (EARP) [35] questionnaire. However, these have been developed largely within the rheumatology community, and their availability use in dermatology clinics is fairly low. Consequently, the detection and management of PsA by dermatologists at the most favorable time remains sub-optimal [36]. Developing the concept of early PsA detection and management within the dermatology clinic needs to be a priority to improve outcomes and facilitate early combined intervention with rheumatology.

### 2.3. Recognising Psoriatic Arthritis

The difficulty for dermatologists in recognizing arthritis in psoriasis patients is compounded by the extremely variable spectrum of musculoskeletal pathologies found in PsA, including clinical entities such as RA-like polyarthritis, ankylosing spondylitis (AS)-like axial disease, isolated enthesitis, oligoarthritis, tendontitis, or arthropathies associated with either bone destruction (arthritis mutilans) or new bone formation (SAPHO syndrome) [4,37]. The unpredictable, heterogeneous and often insidious involvement of the joints and juxta-articular tendons and ligaments in PD can make the distinction from other types of arthritis challenging. Disease classification criteria do exist, but there are currently no diagnostic criteria for PsA, essentially meaning that the diagnosis largely remains one of exclusion of the other arthropathies. To date, the most useful are the CASPAR Criteria (Classification Criteria for Psoriatic Arthritis) [38], which have been validated as a means of classifying PsA for research studies, and have assisted in standardizing the inclusion of patients for clinical trials, with reported sensitivity and specificity of 91.4% and 98.7% respectively in established PsA and of 87.4% and 99.1% respectively in early PsA. However, further validation studies in patients with early PsA (predominantly tenosynovitis/enthesitis/dactylitis pattern) are encouraged, since a disappointingly low sensitivity of only 68.4% for the CASPAR criteria was also reported [39].

**Figure 1:**
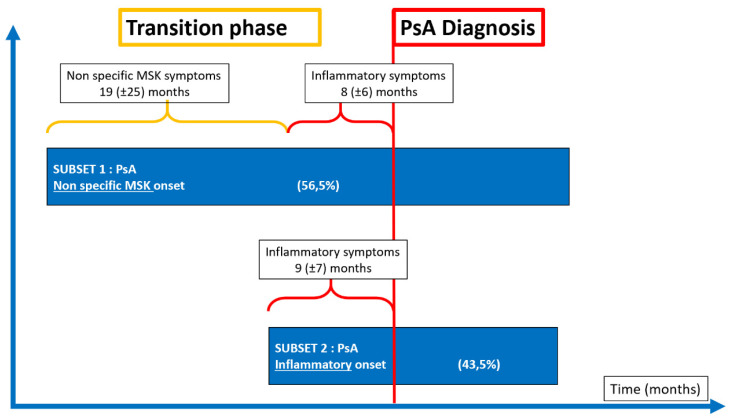
Clinical onset characteristics of 88 patients with a new diagnosis of psoriatic arthritis (PsA), followed in the Early Arthritis Clinic in Udine (Italy), MSK: musculoskeletal.

A typical patient with established PsA will report persistent joint pain, which may or may not be associated with early morning stiffness that lasts for more than thirty minutes (Figure 1). However, the arthralgia may not be as pronounced as in other arthropathies, particularly in the early stages. While virtually 100% of patients with RA and AS, for example, complain of morning stiffness, only about half of PsA patients give the same complaint [40].

Axial involvement in psoriatic arthritis is a well-recognized manifestation with a prevalence between 12.5% and 78% [41]. Inflammatory spondylitis has been reported in a single study to occur in 78% of patients with PsA, although this is typically an imaging diagnosis and is often asymptomatic [41]. Back pain persisting for more than three months, accompanied by morning stiffness for more than half an hour and that is relieved by exercise, is heavily suggestive and should prompt further assessment. This recognition of axial disease is especially clinically challenging.

### 2.4. The Entheseal Inflammation in Early PsA

The key to the recognition of early PsA is entheseal associated inflammation. Although it is difficult to prove the primacy of enthesitis in PsA, several animal models of inflammatory arthritis that manifest as synovitis have shown that the earliest discernable joint abnormality is located at the enthesis, with inflammation subsequently spreading into the adjacent soft tissues [42]. In health, entheses are completely avascular, being fibrocartilaginous in nature at the insertion points and adjacent to the insertion points. The ligament or tendinous structure has tightly cross-linked collagens, which minimizes the degree of vascularity. Consequently, during subtle bouts of inflammation, these tissues do not swell to a significant degree, and the classical features of inflammation may be difficult, or even impossible to discern. Furthermore, age-related thickening of entheses is described [43], and finally, normal entheses are prone to degenerative conditions [23].

As a consequence of enthesis homeostasis and biology, clinical swelling is absent, joint pain and discomfort may be dismissed by the dermatologist as unrelated, and the patient not referred to a rheumatologist, especially if associated with a lack of elevation in the C-reactive protein (CRP) and other inflammatory markers. Whilst the absence of clinical swelling and normal inflammatory markers may be good long-term prognostic signs, if patients are sidelined, the opportunity for early intervention is lost and they may subsequently experience protracted pain and stiffness or declined potentially useful therapies.

### 2.5. Patterns of Joint Involvement

To facilitate recognition of PsA, a thorough joint examination, including foot and ankle, should be incorporated into the baseline dermatological assessment of all patients with PsO, and repeated when symptoms arise. Dermatologists must therefore develop an understanding of the patterns of joint involvement in PsA, although the broad variation and dynamic disease course introduces complexity. What begins as oligoarthritis (four or fewer joints) may convert to polyarticular disease over years, and with treatment may revert back to oligoarthritis. Axial involvement may occur at the onset, develop later in the disease course, or be absent entirely [41]. This explains the difficulty in establishing diagnostic criteria. PsA typically presents with a peripheral arthritis, and thus needs to be distinguished from RA. Table 2 compares the frequency of lesions seen in PsA with RA and inflammatory osteoarthritis (OA). Given the similarities, clinicians cannot rely on patterns of involvement alone to distinguish amongst different forms of arthritis. Furthermore, difficulty is introduced by the fact that PsA often begins without symptoms or clinical signs of joint disease [3,8]. With advances in screening techniques, it is at this early stage that the dermatologist is in an opportune position to detect pre-clinical PsA and make significant impact on the prevention of progressive joint damage [18].

### 2.6. Defining Clinical Disease Phenotypes

It is now quite clear that PsO is a heterogeneous disease with many variants, and some PsO phenotypes sometimes overlap with other disorders such as acneiform lesions (e.g., SAPHO syndrome) and autoinflammatory disease with different genetic predisposition [4]. It has emerged that not all PsO phenotypes are the same, and that certain clinical features are associated with an increased risk of developing PsA. A careful clinical examination of involved sites can therefore provide the dermatologist with an indication of potential future or concurrent subclinical PsA, to which they can target further imaging assessment. Of particular note, it appears that scalp, nail, and gluteal cleft disease are the strongest biomarkers for the development of PsA [26]. These psoriasis patterns are not associated with the carriage of HLA-Cw0602, which is a marker for extensive psoriasis.

This phenotypic heterogeneity may go to some length to explain why some patients arrive at the dermatology clinic with severe PsO but no arthropathy, and conversely, many people with PsA have minimal or even no skin disease. This dichotomy in skin disease phenotypes probably explains why there are such disparate practices amongst rheumatologists and dermatologists.

**Figure 2:**
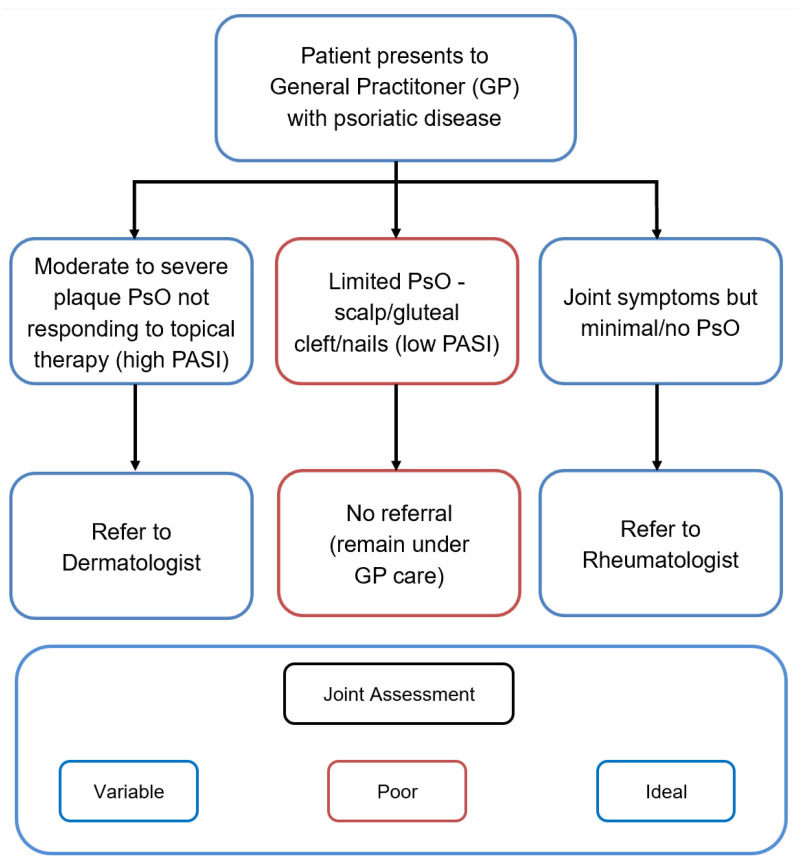
Current practice for psoriatic disease referral to secondary care in the United Kingdom. PsO: Psoriasis; PASI: Psoriasis Area Severity Index.

**Figure 3:**
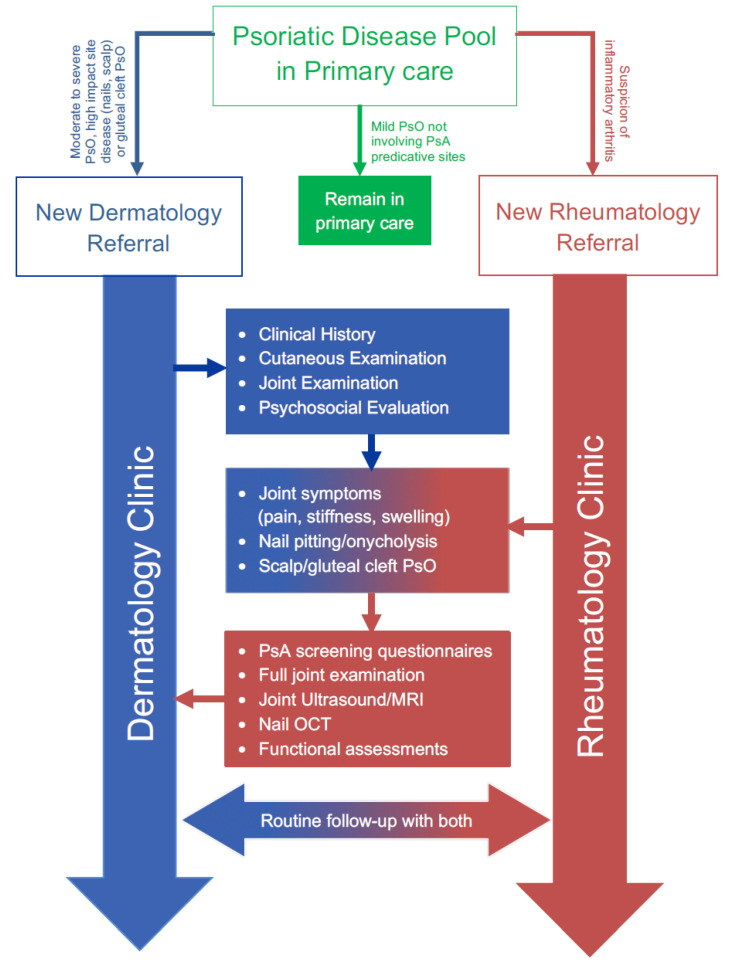
The Leeds Model for shared care in psoriatic disease. MRI: Magnetic Resonance Imaging; OCT: Optical coherence tomography.

At the present time, patients referred for a specialist’s opinion from a dermatologist generally present with psoriasis at the more severe end of the spectrum. Rheumatologists look specifically for any cutaneous signs (skin and/or nails) of psoriasis to aid a diagnosis of early PsA in patients with seronegative arthritis. Primary Care Practitioners (PCPs) are unlikely to refer patients with mild PsO or scalp limited disease to a dermatologist, and may refer to a rheumatologist only when musculoskeletal symptoms develop (Figure 2 and Figure 3). This highlights an unmet educational need for PCPs who see the majority of patients with localized, yet prognostic (i.e., scalp, gluteal cleft, nail) disease, and are thus in a prime position to identify patients with subclinical joint disease. Increased awareness is required in primary care of the need to refer as soon as these cutaneous biomarkers become apparent.

Consequently, in our opinion, it seems judicious that for the optimal management of the spectrum of this condition, clinics with the joint input from both specialties could help for a better definition of the full spectrum of disease. In addition, we believe that there is scope for services that permit for the assessment of patients with low PASI (Psoriasis Area Severity Index) scores, but who exhibit isolated scalp and/or nail disease, as these patients are typically managed within the primary care setting, but could be the group with the greatest burden of underlying skeletal disease [8,26,44].

Optimal detection and understanding of the pre-clincal phases of PsA demands an integrated dermato-rheumatology service to proactively detect key patterns of psoriatic disease. In a research setting, we have shown that psoriasis with nail involvement is associated with a greater burden of subclinical enthesopathy, thus providing a link between nail disease and clinical PsA evolution [44,45].

### 2.7. Combined Dermato-Rheumatology Assessment for Early PsA

Given the challenges in diagnosis of PsA, it would appear that the optimum means of assessing patients for early joint involvement is to have a fully trained rheumatologist work in conjunction with the dermatologists, in dedicated psoriatic disease clinics. This may occur by means of a dedicated rheumatology PsA clinic run simultaneously with the dermatology PsO clinic, with discussion between specialists as required, or with a dedicated rheumatologist actively participating in a joint dermato-rheumatology psoriatic disease clinic. This needs to include patients with milder disease or be linked to a community outreach project for the evaluation of such cases.

A dermato-rheumatology psoriatic disease clinic encourages involved clinicians and researchers to identify predictors of PsA development in PsO patients. It is within this context that seminal imaging studies suggest that PsO patients with more marked subclinical peripheral musculoskeletal inflammatory alterations (such as sonographic detected active enthesitis and synovitis) have a higher risk of progressing to early clinical PsA [20,24].

Furthermore, it has recently been proposed that a combined dermato-rheumatology assessment could also improve a correct diagnosis of early polyarthritis, indeed the differential diagnosis between early seronegative RA and early PsA could be difficult because standard radiographs are often unhelpful, no specific biomarkers are available and minimal cutaneous or nail psoriasis could be unnoticed during the rheumatological examination [46]. In a cohort of early arthritis, a quarter of seronegative polyarthritis initially classified as early seronegative RA, presented cutaneous or nail psoriasis (not reported by the patients and unrecognized on the rheumatological examination) on dermatological evaluation during the combined dermatological-rheumatological visit [46]. These psoriatic findings were very limited and mild on the skin or were isolated on the nails and dermoscopy of the clinically suspected lesions was used to confirm the clinical diagnosis of psoriasis [46,47]. These findings could obviously have important implications for the management and outcome of early arthritis, and the authors suggest that in early seronegative polyarthritis a diagnosis of psoriasis should be excluded through a combined dermatologic-rheumatologic evaluation.

Given that early PsA may present at the onset as a poly-enthesitis without swollen joints, the diagnosis of fibromyalgia, a condition significantly associated with PsO [48] and with a widespread musculoskeletal pain including the entheseal pain, should be ruled out [21]. The sonographic assessment of the peripheral entheses proved to be useful to assist clinicians in the differential diagnosis between PsA and fibromyalgia, since the entheseal sonographic signs of inflammation (i.e., entheseal Doppler signal) are significantly associated with PsA [22,49].

### 2.8. Integrated Care for Psoriatic Disease: Current and Potential Benefits

Anywhere between 5% and 40% of PsO patients have musculoskeletal symptoms, but it is difficult to characterize these clinically. The application of US studies has shown that anywhere between 30% and 50% of patients have subclinical skeletal abnormalities [14,15,50], both subclinical enthesitis and synovitis are frequently detected in psoriatic patients asymptomatic for joint pain. These results are also confirmed with other imaging methods, including abnormal bone uptake on scintigraphy [51,52], abnormal bone uptake on PET scanning [53], and abnormal bone marrow oedema on MRI (Magnetic Resonance Imaging) scanning [16,54]. Historically, the bone abnormalities in PsO were simply thought to be related to the periosteum. However, the advent of MRI and a better understanding of how the enthesis is functionally anchored to the underlying trabecular network (analogous to the anchorage of tree roots to the ground), has led to an understanding of how bone changes are directly linked to enthesopathy.

Recent work has shown that subclinical enthesopathy in PsA has a much higher degree of Power Doppler positivity on ultrasound (a presumed marker of inflammation and angiogenesis), in comparison to the entheseal sites of PsO where these changes are less frequent [20,25,55]. This can be explained by our understanding of the changes to the vasculature, which are a specific, unifying feature of many PD manifestations, and is thus likely to underscore common pathogenic processes. It is not surprising, therefore, that the endothelium, as a therapeutic target in PD, has been proposed in a number of previous studies [56,57].

Power Doppler aside, the grey scale imaging changes detected on ultrasound at the enthesis can be difficult to differentiate from OA. However, those patients with subclinical enthesopathy are more likely to progress to clinically evident PsA, and our data suggests that there may be a march from entheseal thickening, to Doppler change, to actual clinical disease [58,59]. Such collaborative work highlights the importance of research between rheumatologists and dermatologists within the dermatology clinic setting, to further delineate the natural history of the earliest phase of PsA and offers novel imaging biomarkers for arthritis development.

### 2.9. Therapeutics

**Figure 4:**
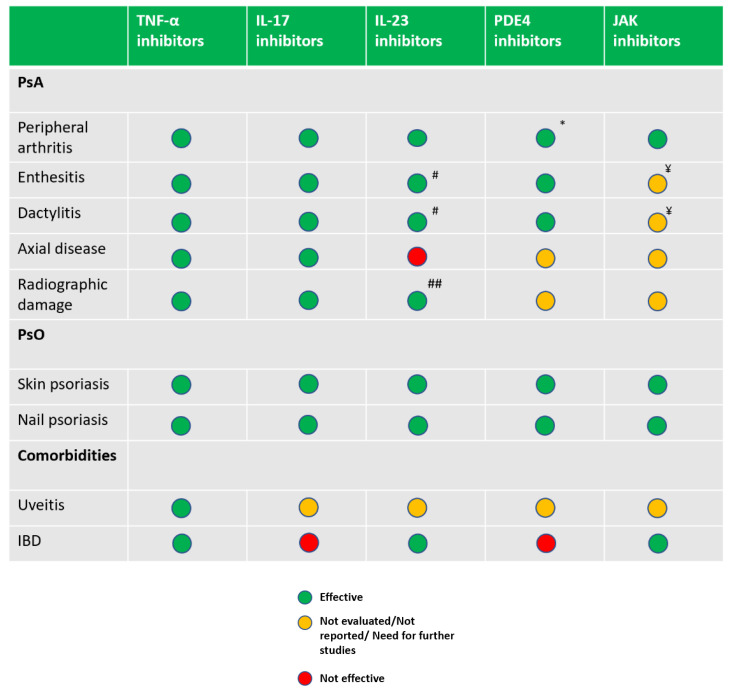
Efficacy of recently introduced therapeutic principles for PsA based on randomised controlled trials stratified for disease domain. ^#^ Risankizumab, no significant difference versus placebo in improving dactylitis and enthesitis [66]. ^##^ Ustekinumab, an anti-IL 12/23 p40 monoclonal antibody, inhibits radiographic progression in patients with active PsA [67]. Guselkumab, an anti-IL23 p19 monoclonal antibody, administered every 8 weeks, resulted in a non-significant decrease in radiographic progression compared with placebo, while Guselkumab every 4 weeks demonstrated significantly less progression of structural damage [68]. * Apremilast is not statistically different compared to placebo in the achievement of ACR70 [69]. ^¥^ With tofacitinib, the changes in scores from baseline with respect to enthesitis and dactylitis could not be declared statistically significant compared to placebo [70].

Combined rheumatology and dermatology psoriasis clinics would merely be of academic relevance, were it not for the fact that there has been a proliferation in the therapeutic armamentarium that is now available to treat both PsO and PsA. Historically, dermatological treatments, including vitamin A and vitamin D analogues and biologic therapies including efalizumab and alefacept, were useful for treating skin disease, but had no proven benefit in joint disease. Likewise, the data for other compounds including ciclosporin is, at the population level, less than convincing for the therapy of PsA [60]. The evidence supporting Methotrexate (MTX) treatment for PsO is strong, while for PsA is controversial. Recommendations by both the European League Against Rheumatism (EULAR) and American College of Rheumatology (ACR) support its use for peripheral arthritis [60,61]. In the recently published Study of Etanercept and Methotrexate in Subjects with Psoriatic Arthritis (SEAM-PsA), the authors described an unexpected high efficacy of MTX monotherapy in enthesitis and dactylitis domain [62]. However, the advent of new systemic treatments, including biologic agents and small molecules, which show efficacy for skin and joint disease, lead dermatologists to ask the question: ”Could the use of treatment for skin disease also control the subclinical arthropathy?” Within the biologic agents, the TNF-α inhibitors and the more recently approved IL-23 and IL-17 inhibitors showed good efficacy both in PsA and PsO, but with different levels of response in the disease domains. IL-23 and IL-17 inhibitors demonstrated a significant superiority for the skin compared to TNF-α inhibitors, while TNF-α inhibitors would still represent the first biological Disease Modifying AntiRheumatic Drugs ( bDMARD) choice in the axial domain [60]. IL-23 inhibitors did not demonstrate efficacy in axial disease [63,64]. In PD, the current role of small molecules is limited, with Apremilast, a phosphodiesterase-4 (PDE4) inhibitor indicated both in PsO and PsA, but recommended in PsA only for mild disease since the overall response rates were relatively low in the studies [60]. At this moment, the only Janus kinases inhibitor (JAKi) approved for PsA, but not for PsO, is tofacitinib, that showed efficacy for peripheral arthritis but need further investigation for the enthesitis and dactylitis domains and for PsO [60,65] (Figure 4).

## 3. Conclusions

Dermatologists are uniquely placed to identify early PsA in patients with psoriatic skin disease, where timely, aggressive intervention should reduce the future health burden of disabling joint damage. It is thus essential that dermatologists develop a clear understanding of the pathogenesis, investigation, and management of PsA. Such an approach can be enhanced through close interaction with rheumatologists, and as our experiences demonstrate, a combined clinic provides the ideal environment for learning, prompt assessment, and immediate discussion as to the optimum management for each individual. This strategy must recognize and incorporate in assessment of community based psoriasis sufferers with only mild skin disease as this group is at particular risk for PsA. For this population of mild PsO, programs of educations for patients and primary care physicians could be crucial for the early recognition of PsA.

The opportunity to pool imaging and nursing resources also confers an economic advantage, and in addition to the savings made from combining two “new” appointments with specialists, there are also those associated with the reduction in the overall cost of caring for patients with debilitating joint disease. With an ever-expanding armamentarium of therapies emerging with efficacy in both PsO and PsA, close liaison between dermatologists and rheumatologists is essential [60,71]. With insufficient data demonstrating efficacy of systemic agents such as methotrexate and ciclosporin, further work is required to ascertain the precise benefits of the biologic therapies in reducing or reversing pre-clincal PsA. Whether differences in PsA prevention or PsA evolution exists between different classes of biologics or conventional DMARDSs awaits further studies. Ultimately, this could translate into improved recognition of early PsA and optimal management of psoriatic disease.

## Figures and Tables

**Table 1:** Pre-clinical markers in early arthritis—psoriatic arthritis versus rheumatoid arthritis.

	Psoriatic Arthritis	Rheumatoid Arthritis
Pre-clinical disease	70% have psoriasis	70% have ACPA positivity
Duration of pre-clinical disease	7–12 years	Up to 10 years
Biomarker/sensitivity	PASI score—no correlation with arthritis severity	ACPA titre—correlates with arthritis severity

ACPA: Anti-citrullinated protein antibodies; PASI: Psoriasis Area Severity Index;

**Table 2:** Comparison of the clinical and biological phenotype of Psoriatic Arthritis (PsA) with that of rheumatoid arthritis (RA) and osteoarthritis OA.

		PsA	RA	Inflammatory OA
Demographics	Male:female	1:1	3:1	1.5:1
	Age at onset	35–45	35–50	45–55
Clinical	Skin Lesions	Frequently	Uncommon	Uncommon
	Nail Lesions	Common	Uncommon	Uncommon
	Dactylitis	Common	Uncommon	Uncommon
	Enthesitis	Common	Uncommon	Uncommon
	DIPJ involvement	Common	Uncommon	Common
	Joint erythema	Common	Uncommon	Uncommon
	Joint tenderness	Mild	Severe	Mild
	Symmetry	Less common	Common	Uncommon
	Back involvement	Common	Uncommon	Uncommon
	Sacroilitis	50%	Rare	Uncommon
Serological	Rheumatoid factor	Uncommon	Common	Uncommon
	ACPA	Uncommon	Common	Uncommon
	HLA-B27	20–30%	Not related	Not related
Imaging	Periostitis	Common	Rare	Uncommon
	Pencil-in-cup	Common	Rare	Uncommon
	Ankylosis	Common	Rare	Uncommon
	Osteopenia	Less common	Common	Uncommon
Morbidity	QoL	Reduced	Reduced	Reduced
	Function	Reduced	Reduced	Reduced

DIPJ: distal interphalangeal joint; HLA: Human Leucocyte Antigen; QoL: Quality of Life.
